# Occurrence of Secondary Malignancies in Chronic Myeloid Leukemia During Therapy with Imatinib Mesylate-Single Institution Experience

**DOI:** 10.4084/MJHID.2015.003

**Published:** 2015-01-01

**Authors:** Grzegorz Helbig, Grażyna Bober, Marek Seweryn, Ryszard Wichary, Andrzej Tukiendorf, Lech Sedlak, Tomasz Oleksy, Sławomira Kyrcz-Krzemień

**Affiliations:** 1 Department of Hematology and Bone Marrow Transplantation, Silesian Medical University, Katowice, Poland.; 2 Department of Statistics, Maria Sklodowska-Curie Memorial Cancer Center and Institute of Oncology, Gliwice, Poland.; 3 Student Research Group, Department of Hematology and Bone Marrow Transplantation, Silesian Medical University, Katowice, Poland.

## Abstract

**Introduction:**

Imatinib mesylate (IM) remains the treatment of choice for chronic myeloid leukemia (CML) showing a remarkable efficacy and offers a perspective for long disease-free survival. Due to prolonged administration of IM, the questions about the possible impact on the development of secondary malignancies (SM) are raised.

**Objective:**

To investigate the incidence and clinical outcome of secondary malignancies during IM therapy for CML.

**Material and Methods:**

The records of 221 CML patients treated with IM between 2003–2013 in a single institution were reviewed. The Poisson regression model was used to estimate the relative risks for SM and death in CML patients.

**Results:**

Secondary malignancies developed in eight out of the 221 patients (3.6%) receiving IM for a median of 61 months (range, 10–137 months). Female/male ratio was 5/3. Two patients were diagnosed with their CML at accelerated phase whereas 6 had chronic phase. The median age at IM initiation was 58 years (range, 31–72 years). Five of these 8 SM patients received IM after other treatments failure: interferon α (n=5), hydroxyurea (n=4) and cytarabine (n=1). Three patients received IM as a frontline therapy. All patients were on IM at 400mg daily at SM occurrence. The therapy for SM included surgery (n=3), chemotherapy only (n=3), and chemotherapy followed by radiotherapy (n=1). One patient did not receive treatment due to disseminated disease. All CML patients were in hematologic and complete cytogenetic response (CCR) at the time of SM development. All of them also met the criteria for major molecular response (BCR-ABL^IS^ ≤0.1%). They continued their IM while receiving treatment for SM. Among eight patients with SM, five patients are alive and remain in CCR on IM whereas three patients died due to SM. The risks for SM development as well as death due to SM in CML patients were not statistically increased if compared to age-adjusted population.

**Conclusions:**

The association between IM therapy for CML and SM development has not been found.

## Introduction

Chronic myeloid leukemia (CML) is a clonal stem cell disorder characterized by the translocation t(9;22)(q34;q11) resulting in creation of the tyrosine kinase chimeric protein BCR-ABL.^1^ Current therapeutic management of CML patients is based on tyrosine kinase inhibitors (TKI). Imatinib mesylate (IM) is a small molecule functioning as a signal transduction inhibitor that specifically targets a set of tyrosine kinase proteins.^2^ This agent is currently used as a treatment of choice for patients with CML showing a remarkable efficacy and providing a perspective for a long disease-free survival.^3^ However, due to the prolonged survival and continuous administration of this agent, the questions about the possible impact on the development of secondary malignancies (SM) are raised. IM was found to possess an immunomodulatory effect on T-cell population as well as dendritic cells changing the immunologic microenvironment.^4^ Data on the possible pathogenic relationship between the development of SM and IM administration are inconclusive and require further investigations.^5^,^6^

Herein, we investigate the occurrence, and clinical outcome of SM in CML patients treated with IM. One of those patients with testicular cancer has been extensively published elsewhere.^7^

## Material and Methods

Two hundred and twenty-one CML patients during IM therapy in our institution between 2003–2013 were included in this analysis. There was population-based material. All patients followed the common standards for CML treatment and cytogenetic/molecular monitoring. Shortly, both cytogenetic and molecular assessments were performed at baseline, then every three months for the first year and every six months thereafter. The records of all included patients were reviewed to assess IM doses, response to therapy and clinical outcome. We divided our CML population into two age groups: 1/≤ 65 and 2/>65 years. The “younger” subgroup included 190 CML patients who developed 7 SM with two deaths due to SM. The “older” group consisted of 31 CML patients who developed 1 SM with fatal outcome.

## Statistical Analysis

The Poisson regression method using WinBUGS software was used to estimate the relative risks for SM and death in CML patients.

## Results

SM were diagnosed in eight out of the 221 patients (3.6%) receiving IM for a median of 61 months (range, 10–137 months). Female/male ratio was 5/3. Two patients were diagnosed with their CML at accelerated phase whereas 6 had chronic phase. The median age at TKI initiation was 58 years (range, 31–72 years). Five of these 8 SM patients received IM after other treatments failure: interferon α (n=5), hydroxyurea (n=4) and cytarabine (n=1). Three patients received IM as a frontline therapy. All patients were on IM at 400mg daily at SM occurrence, and they had no prior history of cancer. All patients were white and developed only one additional cancer. There were eight different malignancies (see table 1).

The therapy for SM included surgery (n=3); chemotherapy only (n=3); and chemotherapy followed by radiotherapy (n=1). One patient did not receive treatment due to disseminated disease. All CML patients were in hematologic and complete cytogenetic response (CCR) at the time of SM development. All of them also met the criteria for major molecular response (BCR-ABL^IS^ ≤0.1%). All patients continued their IM while receiving treatment for their SM. Among eight patients with SM, five patients are alive and remain in CCR on IM whereas 3 patients died due to SM. Following the estimations, no statistical differences between the risks for CML (in the reference population) and the SM (in CML patients) as well as between the age groups (≤65, >65) were established: mean 1.22 [95% CI; 0.14, 4.15], p=0.44 and mean 0.60 [95% CI; 0.09, 1.47], p=0.07, respectively. Moreover, no statistical differences between the risks for the SM (in CML patients) and for death as well as between the age groups (≤65, >65) were also found: mean 1.82 [95% CI; 0.07, 8.33], p=0.47 and mean 3.11 [95% CI; 0.10, 17.2], p=0.41, respectively. In sum, the risks for SM development as well as for death due to SM in CML patients were not statistically increased if compared to age-adjusted population. The summary of SM characteristics in CML subpopulation was shown in table 1.

## Discussion

The efficacy of IM and other TKIs is unquestionable, and early adverse effects are well-known. In general, these drugs are well-tolerated, and most of the side effects are manageable.^3^ Some reports focusing on the oncogenic effect of TKIs have been reported, but the association between the development of SM and TKIs use remains unclear. A 2-year preclinical study in animal models has showed the carcinogenic potential of IM. Papilloma of the preputial and clitoral glands was observed from a dose of 30 mg per kilogram daily that corresponds to a dose of 400 mg used in human beings. Renal, urinary, small intestine, stomach, parathyroid and adrenal gland malignancies were developed at higher IM doses, namely 60 mg/kg/day. Thus, the risk of benign or malignant tumors was found to be increased in the above-mentioned rat models.^8^ However, the potential pathogenic relationship between the development of secondary tumors and IM has not been proved so far.

There are inconsistent reports on the incidence of secondary malignancies in patients with CML treated with TKIs. The first report comes from 2005. The secondary tumors were detected in 6 out of the 189 CML patients treated with IM following IFN failure. The authors suggested an increased incidence of malignant neoplasms among those patients. Especially, the incidence of prostate cancer was found to be four times higher than expected in the population.^5^ In contrast, Pilot et al. performed an epidemiological survey of 9518 CML patients collected in the clinical safety database of Novartis (imatinib manufacturer). In total, this study showed 110 second primary neoplasms and the overall incidence of tumors in this subpopulation was comparable with that of the age-adjusted general population.^6^ Since then, numerous multicenter epidemiological reports have been published. The Imatinib Long Term Effects study detected 30 cases of SM in 832 CML patients with an incidence comparable to the expected. Prostate and breast cancers were the most frequent neoplasms.^3^ Interestingly, a large analysis of 1445 patients with CML and other hematologic malignancies treated with TKIs suggested a lower than expected rate of neoplasms in patients treated with TKIs with observed/expected (O/E) ratio of 0.6. Nevertheless, the incidence of melanoma, endocrine tumors, kidney cancers, and chronic lymphocytic leukemia was higher than expected.^9^

On the other hand, there were several reports demonstrating an increased risk of secondary neoplasms in CML patients receiving TKIs. The retrospective analysis of CML population treated with TKIs in Czech Republic, and Slovakia demonstrated the incidence of secondary malignancies of 3.3%. The prevalence of all malignant tumors except non-melanoma skin cancers was 6.7/1000 person-years, and that is 1.5 times higher than the age-adjusted incidence rate. Median time from the start of TKI therapy to the diagnosis of SM was 32 months.^10^ These data were in line with a report of a German CML study group. A slight increase of SM in CML patients under TKI treatment if compared with the general population has been demonstrated. The most common neoplasms were prostate, colon and lung cancers, as well as non-Hodgkin lymphomas.^11^ Moreover, Japanese authors reported the incidence of secondary neoplasms after TKI therapy to be 16% at ten years in a single institution study that is higher than described in previous reports. It should be mentioned that the tumors developed after a median time of 24 months after TKI administration.^12^ Recently, Shah et al. have published a population study based on The Surveillance, Epidemiology, and End Results (SEER) database to evaluate the incidence of second primary malignancies in CML patients in pre- and post-imatinib eras. It has been shown that the rate of SM in post-imatinib era was significantly higher when compared with pre-imatinib era (O/E ratio 1.48 versus 1.06, respectively). The highest risk of tumor development was found to be within 1–11 months after IM initiation, and a digestive tract was involved the most frequently.^13^ At contrary, a large epidemiological study, based on the Swedish Cancer Registry, found, in imatinib-naïve CML patients, an increased incidence of second neoplasms for stomach, skin, urogenital tract cancers as well as for lymphoid leukemias.^14^ In our study, we did not find an increased risk of SM development. Prior history of sun exposure for melanoma or smoking for bladder cancer was negative. The impact of pre-imatinib treatments on the SM occurrence should be excluded as no strong evidence of their cancerogenic effect does exist. If compared with other studies, the median time from IM initiation to SM detection was longer and exceeded five years.^10^,^13^ We did perform an additional analysis including CML patients treated with second generation TKI and found no secondary malignancies in this study subgroup. It may be due to a lower number of patients treated with second generation TKI if compared with those on imatinib. A shorter duration of therapy/observation may also be involved.

Based on the above-mentioned reports one may ask whether there is an association between IM use and the development of SM. It was demonstrated that this agent has an immunoregulatory effect by inhibiting T-cell activation and proliferation as well as by diminishing the capacity of dendritic cells to elicit primary T-cell responses.^15^ The exposure to IM induces centrosome and chromosome aberrations in cultures of normal human dermal fibroblasts, Chinese hamster embryonal and Indian muntjac fibroblasts in a significant, dose-dependent and species-independent manner. Those aberrant karyotypes emerging under IM use were irreversible after a prolonged culture omitting the drug. Thus, these observations suggest that neoplastic, chromosomally unstable clones may be developed de novo from normal non-hematopoietic cells by IM.^16^ Genetic instability caused by centrosome defects has an important influence in early steps of the development as well as in the progression of many cancers.^17^,^18^,^19^ Moreover, the c-Abl tyrosine kinase was found to promote DNA damage-induced apoptosis. The inhibition of apoptosis associated by TKIs may also explain a proliferative potential of those drugs.^20^

## Conclusions

There is insufficient data to assess that there is an increased risk of developing SM after IM therapy as well as to elucidate the mechanisms through the drug can facilitate carcinogenesis. The “over-risk” of SM occurrence seen in some studies may result from observational bias; CML patients are simply more carefully monitored than the average population. One should consider three different scenarios: 1/a carcinogenic effect of IM therapy, 2) a result of an increased risk of the development of malignancy with ageing in patients with CML, and finally 3) a coincidental occurrence of these two neoplasms in this patient cohort. In sum, it seems reasonable to report all SM that may develop during or after TKIs treatment. Moreover, further molecular studies evaluating carcinogenicity of TKIs would be useful.

## Figures and Tables

**Table 1:** The characteristics of study patients

Patient	Age at diagnosis of CML (years)	Treatment before IM	Age at start of IM (years)	Secondary malignancy (SM)	Site	Interval from start of IM to diagnosis of SM (months)	Outcome and status of SM
1.	49	HU, IFN, Ara-C	52	Adenocarcinoma	endometrium	137	alive; remission after surgery
2.	30	HU, INF	31	Mixed germ cell tumor	testis	14	died; chemoresistance
3.	56	NA	56	Melanoma	skin	10	died; disease dissemination
4.	49	INF	52	Invasive ductal carcinoma	breast	71	alive; on chemotherapy
5.	62	HU, INF	64	Papillary urothelial carcinoma	bladder	51	alive; disease active
6.	60	HU, INF	61	Adenocarcinoma	prostate	84	alive; on chemotherapy
7.	61	NA	61	Adenocarcinoma	large bowel	72	alive; on chemotherapy
8.	72	NA	72	Adenocarcinoma	lung	22	died; disease dissemination

Legend: Ara-C=cytarabine; CML=chronic myeloid leukemia; HU=hydroxyurea; IM=imatinib mesylate; IFN=interferon; NA=non applicable; SM=secondary malignancy
